# Association of frailty status with overall survival in elderly hypertensive patients: based on the Chinese Longitudinal Healthy Longevity Survey

**DOI:** 10.1186/s12889-024-18989-7

**Published:** 2024-05-31

**Authors:** Liying Li, Yueting Liang, Dajun Xin, Lu Liu, Zhuomin Tan, Ziqiong Wang, Muxin Zhang, Haiyan Ruan, Liming Zhao, Kexin Wang, Yi Zheng, Ningying Song, Sen He

**Affiliations:** 1 Department of Cardiology, West China Hospital of Sichuan University, Chengdu, China; 2 Department of Gynaecology and Obstetrics, Karamay Hospital of Integrated Chinese and Western Medicine, Karamay, China; 3 Maternal and Child Health Hospital, Longquanyi District, Chengdu, China; 4 Department of Pharmacology, Shenyang Pharmaceutical University, Shenyang, China; 5 Department of Cardiology, First People’s Hospital, Longquanyi District, Chengdu, China; 6 Department of Cardiology, Traditional Chinese Medicine Hospital of Shuangliu District, Chengdu, China; 7 Department of Cardiology, Hospital of Chengdu Office of People’s Government of Tibetan Autonomous Region, Chengdu, China; 8 Department of Otolaryngology-Head & Neck Surgery, West China Hospital of Sichuan University, Chengdu, China

**Keywords:** Frailty, Elderly, Hypertensive, Overall survival, Accelerated failure time

## Abstract

**Background:**

Hypertension and frailty often coexist in older people. The present study aimed to evaluate the association of frailty status with overall survival in elderly hypertensive patients, using data from the Chinese Longitudinal Healthy Longevity Survey.

**Methods:**

A total of 10,493 elderly hypertensive patients were included in the present study (median age 87.0 years, 58.3% male). Frailty status was assessed according to a 36-item frailty index (FI), which divides elderly individuals into four groups: robustness (FI ≤ 0.10), pre-frailty (0.10 < FI ≤ 0.20), mild-frailty (0.20 < FI ≤ 0.30), and moderate-severe frailty (FI > 0.30). The study outcome was overall survival time. Accelerated failure time model was used to evaluate the association of frailty status with overall survival.

**Results:**

During a period of 44,616.6 person-years of follow-up, 7327 (69.8%) participants died. The overall survival time was decreased with the deterioration of frailty status. With the robust group as reference, adjusted time ratios (TRs) were 0.84 (95% confidence interval [CI]: 0.80–0.87) for the pre-frailty group, 0.68 (95% CI: 0.64–0.72) for the mild frailty group, and 0.52 (95% CI: 0.48–0.56) for the moderate-severe frailty group, respectively. In addition, restricted cubic spline analysis revealed a nearly linear relationship between FI and overall survival (p for non-linearity = 0.041), which indicated the overall survival time decreased by 17% with per standard deviation increase in FI (TR = 0.83, 95% CI: 0.82–0.85). Stratified and sensitivity analyses suggested the robustness of the results.

**Conclusions:**

The overall survival time of elderly hypertensive patients decreased with the deterioration of frailty status. Given that frailty is a dynamic and even reversible process, early identification of frailty and active intervention may improve the prognosis of elderly hypertensive patients.

**Supplementary Information:**

The online version contains supplementary material available at 10.1186/s12889-024-18989-7.

## Background

Hypertension is one of the most prevalent chronic diseases, and it is closely linked to many adverse health outcomes, including cardiovascular disease, chronic kidney disease, and mortality [1, 2]. In 2015, it was estimated that there were 1.13 billion people with hypertension globally [3], and this number is projected to increase to 1.56 billion by 2025 [4]. Particularly, it was estimated that the prevalence of hypertension in people aged > 60 years was over 60% [5], which poses a substantial threat to global public health [1]. Recently, emerging studies have suggested that hypertension and frailty often coexist in the elderly [6, 7], and multiple international guidelines for the management of hypertension have recommended to assess the frailty status in elderly hypertensive patients before initiating antihypertensive medication [8, 9]. Frailty is an age-related geriatric syndrome which differs from disability and illness, and the characteristics of frailty are the physiological reserves of multiple organs and systems decreased and the sensitivity to stressors increased [10, 11], which increases the risk of falls [12], hospitalization [13], fractures [14], and mortality [15] in older people. Frailty and hypertension share some common pathophysiological mechanisms, such as inflammatory response, oxidative stress response [6, 11], they are significantly related and influence each other. On the one hand, frailty increases the risk of hypertension [16, 17]; on the other hand, patients with hypertension have a higher incidence of frailty than those without [18]. However, the causal relationship between them remains unclear.

Frailty has a significant impact on the prognosis of elderly hypertensive patients. A population-based cohort study conducted in the United States revealed that indicators of frailty were associated with an increased risk of serious fall injuries in older hypertensive patients [19]. Based on data from the National Health and Nutrition Examination Survey (1999–2002), Li et al. [20] reported that frailty increased the risk of all-cause mortality threefold in hypertensive participants aged ≥ 65 years. Another study conducted in the United States by Nicholas et al. [21] demonstrated that frailty increased the risk of self-reported falls, injurious falls and all-cause hospitalizations among older hypertensive patients. However, most studies have been implemented in developed countries, and research in developing countries is limited. To our knowledge, only one study from China has filled this gap. A study performed by Ma et al. [22] showed that frailty was associated with a higher 8-year mortality in Chinese hypertensive participants aged ≥ 60 years. However, the population of this study primarily from Beijing, China, and the sample size was relatively small (*n* = 1111), which may limit its generalizability. In addition, few studies explored the dose–response relationship between frailty and prognosis in elderly hypertensive patients.

Therefore, in this study, we used a large nationally representative population to evaluate the association of frailty status with overall survival among Chinese hypertensive patients aged ≥ 65 years.

## Methods

### Study design and participants

Data were obtained from the Chinese Longitudinal Healthy Longevity Survey (CLHLS), a prospective cohort study of community-dwelling older Chinese individuals, which aimed to investigate the factors associated with healthy longevity for older people. The CLHLS was conducted in a randomly chosen half of the counties and cities in 23 of the 31 provinces, covering approximately 85.0% of the Chinese population, and those aged ≥ 80 years accounted for 67.4% of the total participants. The first investigation of this project started in 1998, and follow-up investigations were conducted in 2000, 2002, 2005, 2008, 2011, 2014, and 2018. With a standard questionnaire, well-trained interviewers conducted the survey face to face with participants and collected information of demographic characteristics, socioeconomic characteristics, physical status, psychological status, cognitive function, lifestyles, disease and other information regarding aging. New participants were enrolled during the follow-up to reduce attrition because of loss to follow-up. The study was approved by the Research Ethics Committee of Peking University (IRB00001052-13074), and written informed consent was obtained from each participant. More detailed information of the CLHLS has been described elsewhere [23–25].

**Fig. 1:**
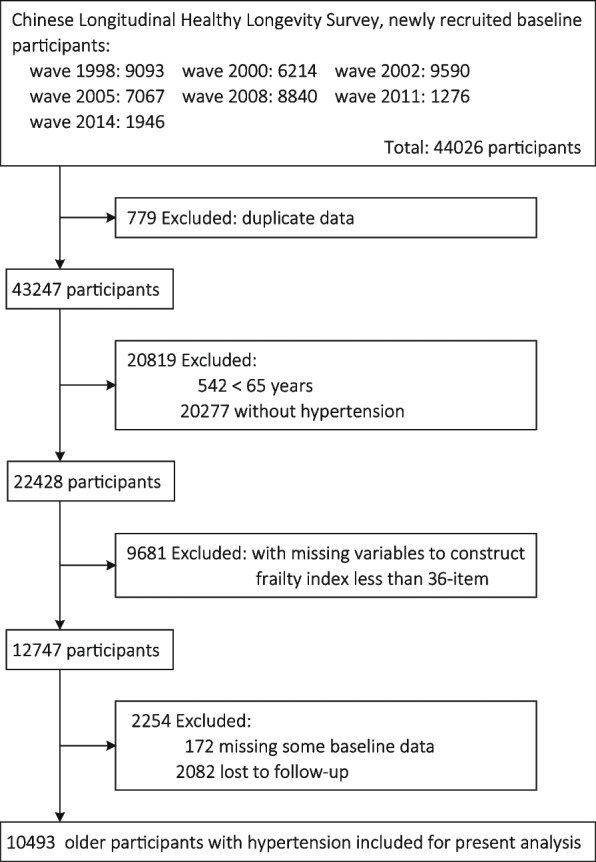
Study flow chart. Note: Except for the missing data, some potential abnormal blood pressure values were defined as missingness, including SBP < 80 or > 300 mm Hg, DBP < 40 or > 200 mm Hg, and SBP minus DBP < 10 or > 200 mm Hg. Abbreviations: SBP = systolic blood pressure; DBP = diastolic blood pressure

The present analysis included seven waves of CLHLS, and the final wave was interviewed during 2018–2019. According to the inclusion and exclusion criteria, 10,493 hypertensive patients aged ≥ 65 years were included in the present analysis. Figure 1 shows the detailed flow chart of inclusion and exclusion process, and Figure S1 shows the spatial distributions of the study population.

### Assessment of hypertension

Single blood pressure (BP) was measured by trained researchers using a mercurial sphygmomanometer after participants had rested for at least 5 min, and hypertension history was assessed by the question “Are you suffering from hypertension?” [26, 27]. Based on the guidelines of the European Society of Hypertension [5], hypertension was defined as systolic BP (SBP) ≥ 140 mm Hg and/or diastolic BP (DBP) ≥ 90 mm Hg or a self-reported history of hypertension. In addition, three waves (2008, 2011, and 2014) provided two BP measurements, and in the sensitivity analyses, the SBP and DBP of an individual were calculated with the average of the two measurements.

### Frailty status assessment

Frailty status was defined by the frailty index (FI), which is one of the extensively used measures of biological age [28]. Based on a standard procedure [29, 30] and available data from all waves of the CLHLS, we modified the 38-item FI [31, 32] appropriately, and the modified FI included 36-item, which encompassed various dimensions of health, including self-reported health, activities of daily living, functional limitations, vision and hearing, diseases and others. The FI counts the cumulative health deficits of a person, items with binary option were scored as 1 (present) or 0 (absence) and with ordinal options were assigned different scores (e.g.: always = 0, often = 0.25, sometimes = 0.5, seldom = 0.75, never = 1) [32]. The 36-item used to construct FI are shown in Table S1, and the corresponding scores of the items are defined in detail. The total deficit score of 36 items divided by 36 is the FI for each participant [31]. The FI ranged from 0 to 1, and the FI distribution of the study population is shown in Figure S2. Participants were divided into four groups according to FI cut-off value in previous study [33]: robustness (FI ≤ 0.10), pre-frailty (0.10 < FI ≤ 0.20), mild-frailty (0.20 < FI ≤ 0.30) and moderate-severe frailty (FI > 0.30).

### Covariates

Covariates were obtained using a structured questionnaire, including age, sex, marital status, residence, co-residence, education, lifestyles (whether smoking, drinking, and regular exercise at present), intake of foods (how often intake of fruit, vegetables, meat, fish, eggs, and beans), and comorbidities (whether suffering from diabetes, heart diseases, cerebrovascular diseases, respiratory diseases, and cancer). Detailed information about the reclassifications of the baseline variables used in the present study is shown in Table S2. More detailed information about these covariates can be found at https://agingcenter.duke.edu/CLHLS. Potential confounding variables associated with hypertension and frailty were adjusted in our analyses.

### Study outcome

The study outcome was overall survival time, defined as the time from baseline to any cause of death. All participants were followed up from the first evaluation up to death or the most recent evaluation. The survival status and date of death of the participants were recorded during the follow-up period of each wave. Participants who could not be contacted after baseline interview were regarded as lost to follow-up. In the present analysis, we defined the maximum follow-up duration as 10 years.

### Statistical analysis

Table S3 shows the distributions of variables with missing data, and the missing values of all baseline variables in the present study were no more than 0.63%. We excluded participants with missing baseline variables in the main analyses. The analyses of the present study include five steps: (1) comparison of baseline data; (2) evaluating the association of frailty status with overall survival in elderly hypertensive patients; (3) exploring the dose–response relationship between FI and overall survival in elderly hypertensive patients; (4) stratified analyses in different subgroups to evaluate the robustness of the main findings; and (5) sensitivity analyses from different perspectives to confirm the stability of the main analysis.

For categorical variables, p value for trend was computed from the Mantel–Haenszel test. For continuous variables, p value for trend across the four groups was computed from the Spearman test when the row-variable was non-normal distribution. Categorical variables are presented as number (percentage) and continuous variables were presented as median (interquartile range [IQR]). Kaplan − Meier analysis was used to estimate the overall survival probability in each group, and difference between the four groups was compared using the log-rank test. Because the variables did not satisfy the proportional hazards assumption of Cox proportional hazards models, a parametric accelerated failure time (AFT) model was used to evaluate the association of frailty status with overall survival, and three adjusted models were constructed. The AFT model directly regresses the logarithm of survival time, and the time ratio (TR) reflects the impact of a variable on survival time. Applying the AFT model to perform survival analysis, a TR > 1 indicated that the survival time was prolongs compared with the reference group, and a TR < 1 indicated that the survival time was shorter than the reference group [34]. The Weibull distribution was selected for AFT models in our analysis based on the minimum Akaike Information Criterion among different survival distributions (e.g.: Weibull, logistic, log-logistic, log-normal, exponential, and Gaussian) (Table S4). We also explored the potential dose–response relationship between FI and overall survival of elderly hypertensive patients using restricted cubic spline (RCS) analysis.

Additionally, stratified analyses were performed to assess the consistency of the association of frailty status with overall survival in different subgroups, and interactions were examined by likelihood ratio testing. Furthermore, a series of sensitivity analyses were performed to assess the robustness of the main findings, including: (1) excluding the participants who died within the first year and the first two years of follow-up to reduce potential reverse causation; (2) mitigating potential bias caused by missing data by performing multiple imputation for the covariate data and then conducting sensitivity analysis; (3) clarifying the role of participants lost to follow-up in the associations of frailty status and overall survival, sensitivity analyses were performed after considering the losses censored occurred at two time points: median (3.38 years) and the end of follow-up time (10.00 years); (4). For three waves (2008, 2011, and 2014 wave) which provided two BP measurements, the SBP and DBP of an individual were calculated with the mean value of the two measurements and then performed sensitivity analyses.

We used R software version 4.1.3 for statistical analyses and a two- sided *p* < 0.05 was considered statistically significant.

## Results

### Baseline characteristics

The baseline characteristics are shown in Table 1. The median age of the participants was 87.0 (IQR: 80.0, 95.0) years, and 6122 (58.3%) participants were men. There were 3346, 4924, 1587, and 636 participants in the robustness group, pre-frailty group, mild-frailty group and moderate-severe frailty group, respectively. The severity of frailty status increased with age, and women had a higher proportion to develop frailty than men. The proportion of elderly hypertensive patients who not in marriage, without received school education and with other comorbidities (diabetes, heart diseases, cerebrovascular diseases, respiratory diseases, and cancer) increased gradually in the pre-frailty, mild-frailty and moderate-severe frailty group. Older hypertensive patients who exercise regularly, and intake of fruit, vegetables, meat, fish, eggs and beans regularly have less probability to develop frailty. The four groups also have significant differences in current smoking, current drinking, SBP, and DBP.

**Table 1:** Baseline characteristics

Variables	All	Frailty status				*p* for trend^a^
		Robustness	Pre-frailty	Mild frailty	Moderate-severe frailty	
Number of participants	10,493	3346	4924	1587	636	
Sex: male	6122 (58.3%)	2247 (67.2%)	2817 (57.2%)	772 (48.6%)	286 (45.0%)	< 0.001
Age (years)	87.0 (80.0, 95.0)	82.0 (75.0, 90.0)	88.0 (81.0, 95.0)	92.0 (85.0, 100.0)	95.0 (89.0, 100.0)	< 0.001
Marital status						< 0.001
In marriage	3447 (32.9%)	1469 (43.9%)	1501 (30.5%)	359 (22.6%)	118 (18.6%)	
Not in marriage	7046 (67.1%)	1877 (56.1%)	3423 (69.5%)	1228 (77.4%)	518 (81.4%)	
Residence						0.177
Urban	4519 (43.1%)	1435 (42.9%)	2087 (42.4%)	711 (44.8%)	286 (45.0%)	
Rural	5974 (56.9%)	1911 (57.1%)	2837 (57.6%)	876 (55.2%)	350 (55.0%)	
Co-residence						0.694
With household members	8806 (83.9%)	2739 (81.8%)	4157 (84.4%)	1356 (85.5%)	554 (87.1%)	
Alone	1275 (12.2%)	541 (16.2%)	546 (11.1%)	145 (9.1%)	43 (6.8%)	
In an institution	412 (3.9%)	66 (2.0%)	221 (4.5%)	86 (5.4%)	39 (6.1%)	
Education						< 0.001
1 year or more	4615 (44.0%)	1768 (52.8%)	2074 (42.1%)	576 (36.3%)	197 (31.0%)	
No school	5878 (56.0%)	1578 (47.2%)	2850 (57.9%)	1011 (63.7%)	439 (69.0%)	
Lifestyles						
Current smoking	2442 (23.3%)	1047 (31.3%)	1088 (22.1%)	250 (15.8%)	57 (9.0%)	< 0.001
Current drinking	2687 (25.6%)	1077 (32.2%)	1243 (25.2%)	287 (18.1%)	80 (12.6%)	< 0.001
Current regular exercise	3528 (33.6%)	1411 (42.2%)	1708 (34.7%)	355 (22.4%)	54 (8.5%)	< 0.001
Regular intake of foods						
Fruit	2805 (26.7%)	1085 (32.4%)	1210 (24.6%)	364 (22.9%)	146 (23.0%)	< 0.001
Vegetables	8750 (83.4%)	2948 (88.1%)	4120 (83.7%)	1242 (78.3%)	440 (69.2%)	< 0.001
Meat	3907 (37.2%)	1452 (43.4%)	1741 (35.4%)	521 (32.8%)	193 (30.4%)	< 0.001
Fish	2151 (20.5%)	794 (23.7%)	988 (20.1%)	268 (16.9%)	101 (15.9%)	< 0.001
Eggs	4144 (39.5%)	1445 (43.2%)	1880 (38.2%)	574 (36.2%)	245 (38.5%)	< 0.001
Beans	3502 (33.4%)	1260 (37.7%)	1591 (32.3%)	455 (28.7%)	196 (30.8%)	< 0.001
Comorbidities						
Diabetes	202 (1.9%)	19 (0.6%)	90 (1.8%)	54 (3.4%)	39 (6.1%)	< 0.001
Heart disease	958 (9.1%)	84 (2.5%)	437 (8.9%)	297 (18.7%)	140 (22.0%)	< 0.001
Cerebrovascular diseases	513 (4.9%)	24 (0.7%)	177 (3.6%)	160 (10.1%)	152 (23.9%)	< 0.001
Respiratory disease	1261 (12.0%)	110 (3.3%)	688 (14.0%)	312 (19.7%)	151 (23.7%)	< 0.001
Cancer	47 (0.5%)	2 (0.1%)	22 (0.5%)	10 (0.6%)	13 (2.0%)	< 0.001
SBP (mm Hg)	150.0 (140.0, 160.0)	146.0(140.0, 160.0)	150.0(140.0, 160.0)	150.0(140.0, 165.0)	150.0 (140.0, 160.5)	< 0.001
DBP (mm Hg)	90.0 (80.0, 95.0)	90.0 (80.0, 92.0)	90.0 (80.0, 95.0)	90.0 (80.0, 96.0)	90.0 (80.0, 96.0)	< 0.001

Values are median (interquartile range) or n (percentage)

*Abbreviations*: *SBP* systolic blood pressure, *DBP* diastolic blood pressure

^a^For these groups: robustness, pre-frailty, mild frailty and moderate-severe frailty

### Association of frailty status with overall survival

**Fig. 2:**
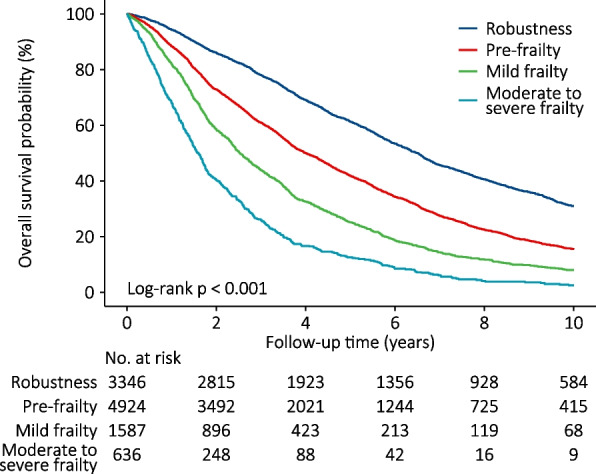
Association of frailty status with overall survival in elderly hypertensive patients by Kaplan–Meier survival curves

During a period of 44,616.6 person-years of follow-up, 7327 (69.8%) participants died. The mortality rates gradually increased from the robustness group to the moderate-severe frailty group, and the rates were 10.6 per 100 person-years (95% CI: 10.1–11.0), 17.5 per 100 person-years (95% CI: 17.0–18.0), 26.3 per 100 person-years (95% CI: 25.1–27.5), and 41.9 per 100 person-years (95% CI: 39.3–44.5) for the robustness group, the pre-frailty group, the mild-frailty group, and the moderate-severe frailty group, respectively (Table 2). Kaplan − Meier analysis revealed a gradual decrease in overall survival probability among elderly hypertensive patients in the robustness, pre-frailty, mild-frailty and moderate-severe frailty groups (log- rank *p* < 0.001) (Fig. 2). In the AFT analysis, compared to those in the robustness group, the unadjusted TRs were 0.63 (95% CI: 0.60–0.66, *p* < 0.001), 0.43 (95% CI: 0.41–0.46, *p* < 0.001), and 0.28 (95% CI: 0.26–0.30, *p* < 0.001) in the pre-frailty group, mild-frailty group and moderate-severe frailty group, respectively. After adjusting for sex, age, marital status, residence, co-residence, education, lifestyles, regular intake of foods, SBP and DBP, the TRs were 0.84 (95% CI: 0.80–0.87, *p* < 0.001) in the pre-frailty group, 0.68 (95% CI: 0.64–0.72, *p* < 0.001) in the mild-frailty group and 0.52 (95% CI: 0.48–0.56, *p* < 0.001) in the moderate-severe frailty group (Table 2), which means the overall survival time of those with pre-frailty, mild-frailty and moderate-severe frailty was reduced by 16%, 32% and 48% compared with the robustness elderly. Moreover, multivariate AFT analysis revealed that the overall survival time of elderly hypertensive patients gradually decreased with the deterioration of frailty status (*p* for trend < 0.001) (Table 2).

**Table 2:** Associations of frailty status with overall survival in hypertensive patients

	Frailty status				*p *for trend^b^
	Robustness	Pre-frailty	Mild frailty	Moderate-severe frailty	
Number of participants	3346	4924	1587	636	
Number of deaths	1905	3542	1301	579	
Follow-up (PYs)	18,025.5	20,258.6	4949.8	1382.7	
Mortality rates^a^ (95% CI)	10.6 (10.1–11.0)	17.5 (17.0–18.0)	26.3 (25.1–27.5)	41.9 (39.3–44.5)	
Unadjusted TR (95% CI), p	1.00 (ref)	0.63 (0.60–0.66), < 0.001	0.43 (0.41–0.46), < 0.001	0.28 (0.26–0.30), < 0.001	< 0.001
Adjusted TR (95% CI), p					
Model 1	1.00 (ref)	0.82 (0.78–0.86), < 0.001	0.66 (0.62–0.70), < 0.001	0.49 (0.46–0.53), < 0.001	< 0.001
Model 2	1.00 (ref)	0.83 (0.79–0.86), < 0.001	0.66 (0.63–0.70), < 0.001	0.50 (0.46–0.54), < 0.001	< 0.001
Model 3	1.00 (ref)	0.84 (0.80–0.87), < 0.001	0.68 (0.64–0.72), < 0.001	0.52 (0.48–0.56), < 0.001	< 0.001

Model 1 with adjustment for sex and age

Model 2 with adjustment for variables in model 1 plus marital status, residence, co-residence, education, systolic BP and diastolic BP

Model 3 with adjustment for variables in model 2 plus lifestyles (current smoking, current drinking, current regular exercise), and regular intake of foods (fruit, vegetables, meat, fish, eggs, beans)

*Abbreviations*: *PYs* person-year, *TR* time ratio, *CI* confidence interval, *FI* frailty index, *BP* blood pressure

^a^Per 100 person-years

^b^Test for trend on variable containing median value of FI for each group

### Dose–response relationship between FI and overall survival

**Fig. 3:**
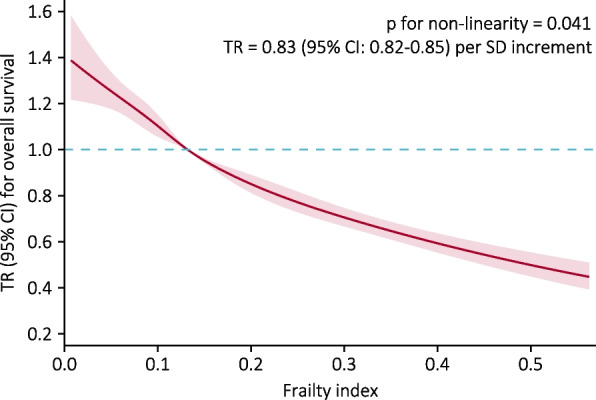
Dose–response relationship between frailty index and overall survival in elderly hypertensive patients. Note: The frailty index was modeled using a restricted cubic spline with five knots at the 5th, 27.5th, 50th, 72.5th and 95th percentiles. TR and 95% CI were derived from AFT model with adjustment for sex, age, marital status, residence, co-residence, education, lifestyles (current smoking, drinking, and regular exercise), intake of foods (fruit, vegetables, meat, fish, eggs, and beans), systolic BP and diastolic BP. Abbreviations: TR = time ratio; CI = confidence interval; SD = standard deviation; BP = blood pressure

The potential dose–response relationship between FI and overall survival was explored by RCS analysis, and adjusted RCS analysis demonstrated a nearly linear relationship between FI and overall survival (p for non-linearity = 0.041) (Fig. 3). The adjusted TR gradually decreased with the increase of FI. After adjusting for potential confounding variables, the overall survival time decreased by 17% with per standard deviation (SD) increase in FI (TR = 0.83, 95% CI: 0.82–0.85) (Fig. 3).

### Stratified analysis

**Fig. 4:**
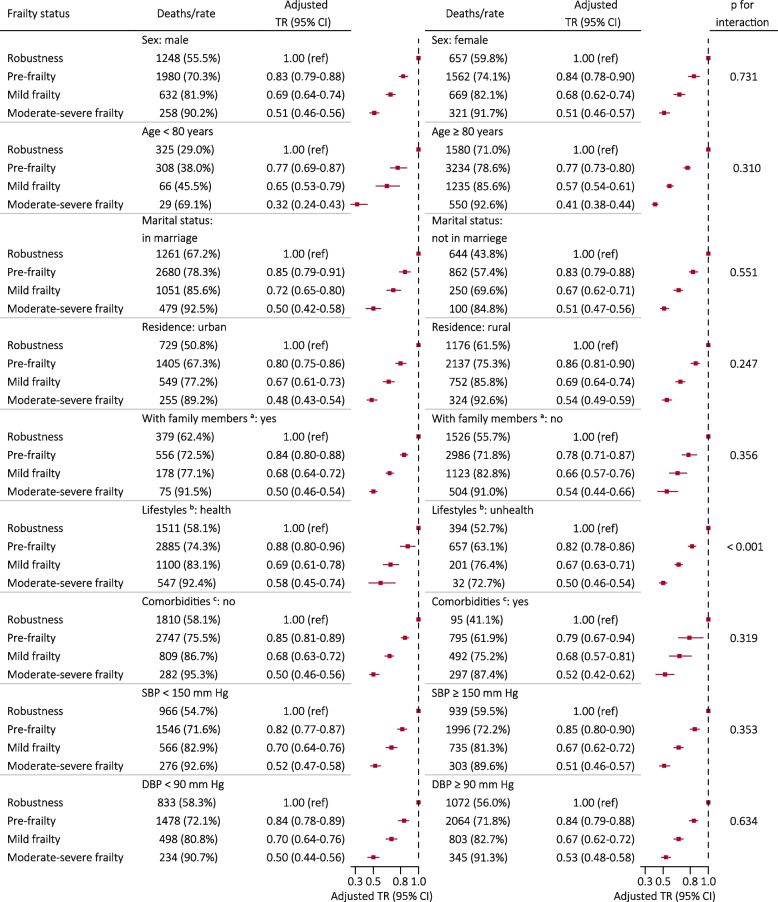
Stratified analyses by potential modifiers of the association between frailty status and overall survival. Note: ^a^If the co-residence of a participant was “with household members”, he/she was defined as “with family members: yes”, and if the co-residence was “alone” or “in an institution”, he/she was defined as “with family members: no”; ^b^If a participant met all the criteria, including current smoking (no), current drinking (no), and current regular exercise (yes), he/she was defined as “healthy lifestyles”; otherwise, he/she was defined as “unhealthy lifestyles”. ^c^If a participant was without any comorbidity in the Table 1, he/she was defined as “no”; otherwise, he/she was defined as “yes”. Each stratification adjusted for sex, age, marital status, residence, co-residence, education, lifestyles (current smoking, current drinking, current regular exercise), regular intake of foods (fruit, vegetables, meat, fish, eggs, beans), SBP and DBP, except for the stratification factor itself. Abbreviations as in Table 1

Stratified analysis revealed a gradual decrease in overall survival time among elderly hypertensive patients as frailty status increased (Fig. 4). Frailty exerted a more pronounced impact on overall survival time in elderly hypertensive participants with unhealthy lifestyles (p for interaction < 0.001), but it did not differ by sex, age, marital status, residence, with family members or not, SBP and DBP (Fig. 4).

### Sensitivity analysis

A series of sensitivity analyses were performed to assess the robustness of the main findings. The impact of frailty status on the overall survival was not significantly changed after excluding participants who died within the first year or the first two years of follow-up (Table S5). After multiple imputation for the missing data of baseline variables, the results showed that the independent association between frailty status and overall survival was preserved (Table S6). Moreover, the findings remained consistent after we regarded participants lost to follow-up as censored at the time of median (3.38 years) or the end of follow-up (10.00 years) (Table S7). In addition, consistent results were also observed when BP was calculated with the average of the two measurements of the three waves (2008, 2011, and 2014) (Table S8).

## Discussion

Based on a national prospective cohort study, we investigated the association between frailty status and overall survival in elderly Chinese hypertensive participants. The results of our study revealed that the overall survival time of elderly hypertensive patients gradually decreased with the deterioration of frailty status. We also explored the dose–response relationship between FI and overall survival, and the findings demonstrated a nearly linear relationship between FI and overall survival time.

The prevalence of hypertension and frailty increases with advancing age, and both are main risk factors for mortality in older people [2, 10]. With the aging of the population, hypertension and frailty impose a substantial burden on healthcare systems [1, 10]. Frailty and hypertension often coexist in older people, frailty increases the risk of hypertension in the elderly [16, 17], while older people with hypertension are more likely to be frail than the general population [18]. Prior research has demonstrated that frailty is a risk factor for the prognosis of elderly patients with hypertension. An analysis of data from the Beijing Longitudinal Study of Aging (2004–2012) used 68-item FI to assess frailty, and the result showed that frailty was associated with a higher risk of all-cause mortality in older people with hypertension (adjusted hazard ratio [HR] = 2.16) [22]. A population-based cohort study from the United States included 5236 hypertensive patients aged ≥ 65 years, and it used six indictors (low body mass index, cognitive impairment, depressive symptoms, exhaustion, impaired mobility, and history of falls) to assess frailty. The results demonstrated that indicators of frailty were associated with an increased risk of serious fall injuries, compared to those without frailty indicators, the adjusted HRs for a serious fall injury in participants with 1, 2, or ≥ 3 indicators of frailty were 1.18 (95% CI: 0.99–1.40), 1.49 (95% CI: 1.19–1.87), and 2.04 (95% CI:1.56–2.67), respectively [19]. Li et al. [20] used data from the National Health and Nutrition Examination Survey (1999–2002) to explore the association of frailty with all-cause mortality. In this study, they used five criteria (weakness, exhaustion, low physical activity, shrinkage, and slowness) to assess frailty, and the results showed that pre-frailty and frailty were associated with an increased risk of all-cause mortality in older hypertensive participants aged 65 years or older. It is worth noting that the definition of frailty is different in these studies, and the main reason is that there is no unified standard of frailty definition in the international. Currently, the Fried phenotype and FI are the two most widely used tools for frailty assessment. We used FI to define frailty, and the findings of our study provide further evidence supporting the impact of frailty on the prognosis of elderly patients with hypertension. We observed a significant decrease in overall survival time among elderly hypertensive patients with the increase severity of frailty status, compared with those robustness elderly, the overall survival time of elderly hypertensive with pre-frailty, mild-frailty and moderate-severe frailty was reduced by 16%, 32% and 48%. In the stratified analysis, we found that frailty had a more pronounced impact on the overall survival time of elderly hypertensive patients with unhealthy lifestyles (smoking, drinking and lacking of regular exercise). The possible reason is that these unhealthy lifestyles are risk factors for frailty, hypertension and many other diseases, leading to further deterioration in the health status of elderly patients, and then increasing the risk of adverse outcomes. Thus, we should advocate and encourage older people to exercise regularly, and not to smoke and drink.

Although FI has been used to evaluate frailty status of the elderly in many studies, few studies have explored dose–response relationship between FI and prognosis in older people with hypertension. To our knowledge, only one study from the United States [21] explored dose–response relationship between FI and prognosis in older hypertensive patients, which revealed that per 1% increase in FI, the risk of self-reported falls, injurious falls and all-cause hospitalizations increased by 3%, 3.5% and 3.8%, respectively. In the present study, the potential dose–response relationship between FI and overall survival of elderly hypertensive patients was explored by RCS analysis, and a nearly linear relationship was observed between them and it indicated that the overall survival time of older hypertensive patients was reduced by 17% with per SD increase in FI.

Frailty not only affects the prognosis of elderly patients with hypertension, it also has an important impact on the prognosis of the general population. A cohort study from the United Kingdom [35] included 493,737 participants aged 37–73 years, the results showed that pre-frailty and frailty increased the risk of mortality in women aged 45–73 years and in men aged 37–73 years. It also indicated the prevalence of frailty increased with age, and participants with frailty were more likely to be female, which is consistent with our findings.

The precise mechanisms that frailty increases the risk of adverse outcomes in elderly hypertensive patients remain unclear, maybe it can be explained by the following ways. First, frail elderly hypertensive patients often present with multiple comorbidities, leading to polypharmacy [36], which may potentially increase the risk of adverse events that associated with drug reactions and drug interactions in older people [37]. Second, frailty has a negative influence on the adherence to antihypertensive treatment among elderly hypertensive patients [38], thereby impeding effective management of BP, and then increase the risks of hypertension-related adverse events. Third, the significance of frailty is frequently overlooked in clinical practice, and the failure of doctors to assess the frailty status in older adults prior to initiating antihypertensive therapy may lead to overtreatment, increasing the risk of hypotension-related events. In addition, frailty is correlated with inflammation and oxidative stress, which may exacerbate cardiovascular disease in patients with hypertension and then increase the risk of cardiovascular events [6, 39].

Frailty changes dynamically over time and it is partially reversible [28, 40], therefore, it is important to identify frailty at an early stage and take appropriate interventions actively to prevent the deterioration of frailty. Physical activity, psychosocial support, healthy lifestyles, management of multimorbidity and chronic diseases, control of weight, intake of protein and micronutrient deficits could improve frailty status [28, 41].

To our knowledge, this is the first study to analyze the impact of frailty status on overall survival in Chinese hypertensive patients aged ≥ 65 years. Our study has some strengths: on the one hand, the CLHLS is a prospective and population-based cohort study that covers most provinces and cities of China, and the present study had a considerably large sample size and a relatively long follow-up period; on the other hand, we utilized a health deficit indicator, a more comprehensive frailty assessment tool, which was constructed following a standard procedure [29, 30], to assess the frailty status of older hypertensive participants. However, this study also exists several limitations. First, information was collected using a standard questionnaire, some of the data may exist recall bias for participants. Second, BP was measured only once in some waves, however, it was measured by a well-trained research assistant using a mercurial sphygmomanometer after participants had at least 5 min of rest, which means the BP is reliable. In addition, we also calculated the mean SBP and DBP of three waves (2008, 2011, 2014) for further sensitivity analyses, and the results are similar to the main findings. Third, although possible confounders were adjusted as much as possible in our analyses, some residual and unmeasured confounding variables may still exist. Fourth, this study only included people aged ≥ 65 year, therefore, the findings cannot be generalized to younger populations. Finally, we only evaluated the association of baseline frailty status and overall survival in elderly hypertensive patients, the association of changes in frailty status with overall survival was not determined.

## Conclusions

The overall survival time of elderly hypertensive patients decreased with the deterioration of frailty status. Given that frailty is a dynamic and even reversible process, early identification of frailty and active intervention may improve the prognosis of older people with hypertension.

### Supplementary Information

Supplementary Material 1.

## Data Availability

Researchers can download the datasets free of charge from the following websites: (1) https://opendata.pku.edu.cn; Peking University Open Access Research Database; (2) https://www.icpsr.umich.edu/icpsrweb/NACDA/series/487; National Archive of Computerized Data on Aging (NACDA) sponsored by U.S. National Institute of Aging (NIA/NIH), Inter-university Consortium for Political and Social Research (ICPSR) at University of Michigan.

## Abbreviations

FI: Frailty index
TR: Time ratio
CI: Confidence interval
CLHLS: Chinese Longitudinal Healthy Longevity Survey
BP: Blood pressure
SBP: Systolic blood pressure
DBP: Diastolic blood pressure
IQR: Interquartile range
AFT: Accelerated failure time
RCS: Restricted cubic spline
SD: Standard deviation
HR: Hazard ratio
