# Supplementary material for: Association of frailty status with overall survival in elderly hypertensive patients: based on the Chinese Longitudinal Healthy Longevity Survey
Source: BMC Public Health. 2024 May 31;24:1468. doi: 10.1186/s12889-024-18989-7 (PMC11143568; doi:10.1186/s12889-024-18989-7)

Association of frailty status with overall survival in elderly hypertensive patients: based on the Chinese Longitudinal Healthy Longevity Survey

Liying Li[1], Yueting Liang[2], Dajun Xin[3], Lu Liu[1], Zhuomin Tan[4], Ziqiong Wang[1], Muxin Zhang[5], Haiyan Ruan[1,6], Liming Zhao[7], Kexin Wang[1], Yi Zheng[1], Ningying Song[8,*], Sen He[1,*]

(Liying Li, Yueting Liang, and Dajun Xin equally contribute to the article)

1. Department of Cardiology, West China Hospital of Sichuan University, Chengdu, China
2. Department of Gynaecology and Obstetrics, Karamay Hospital of Integrated Chinese and Western Medicine, Karamay, China
3. Maternal and Child Health Hospital, Longquanyi District, Chengdu, China
4. Department of Pharmacology, Shenyang Pharmaceutical University, Shenyang, China
5. Department of Cardiology, First People's Hospital, Longquanyi District, Chengdu, China
6. Department of Cardiology, Traditional Chinese Medicine Hospital of Shuangliu District, Chengdu, China
7. Department of Cardiology, Hospital of Chengdu Office of People's Government of Tibetan Autonomous Region, Chengdu, China
8. Department of Otolaryngology-Head & Neck Surgery, West China Hospital of Sichuan University, Chengdu, China

[*]Corresponding author:

Ningying Song: Department of Otolaryngology-Head & Neck Surgery, West China Hospital of Sichuan University, Chengdu, China; E-mail: songningying2@163.com

Sen He: Department of Cardiology, West China Hospital of Sichuan University, Chengdu, China; E-mail: hesen_sky@scu.edu.cn

List of Supplementary Materials

Table S1. Items used to construct the frailty index 3
Table S2. Baseline variable definitions in the present study 5
Table S3. Distributions of baseline variables with missing data 7
Table S4. Akaike information criterion values for parametric models 7
Table S5. Associations of frailty status with overall survival in hypertensive patients after excluding deaths within the first year or first two years 8
Table S6. Associations of frailty status with overall survival in hypertensive patients after multiple imputation (n = 10626) 9
Table S7. Associations of frailty status with overall survival in hypertensive patients in considering the losses censored at the two time points: median (3.38 years) and the end of follow-up (10.00 years) (n = 12575) 10
Table S8. Associations of frailty status with overall survival in hypertensive patients with two BP measurements (n=1164) 11

Figure S1. The spatial distributions of the study population 12
Figure S2. Distributions of frailty index among the study participants 12

Table S1. Items used to construct the frailty index

No.	Variables	Data type	Corresponding score
1	Self-reported health	Ordinal	Very good = 0, good = 0.25, so so = 0.5, bad = 0.75, very bad = 1
2	Feel fearful or anxious	Ordinal	Always = 1, often = 0.75, sometimes = 0.5, seldom = 0.25, never = 0
3	Feel useless with age	Ordinal	Always = 1, often = 0.75, sometimes = 0.5, seldom = 0.25, never = 0
4	Look on the bright side of things	Ordinal	Always = 0, often = 0.25, sometimes = 0.5, seldom = 0.75, never = 1
5	Make own decisions	Ordinal	Always = 0, often = 0.25, sometimes = 0.5, seldom = 0.75, never = 1
6	Keep my belongings neat and clean	Ordinal	Always = 0, often = 0.25, sometimes = 0.5, seldom = 0.75, never = 1
7	Housework at present	Ordinal	Almost every day = 0; not daily, but once for a week =0.25; not weekly, but at least once for a month/sometimes = 0.5; not monthly, but sometimes = 0.75; never = 1
8	ADLs: bathing	Ordinal	Without assistance = 0, one part assistance = 0.5, more than one part assistance = 1
9	ADLs: dressing	Ordinal	Without assistance = 0, need assistance for trying shoes = 0.5, assistance in getting clothes and getting dressed = 1
10	ADLs: toileting	Ordinal	Without assistance = 0, assistance in cleaning or arranging clothes = 0.5, don't use toilet = 1
11	ADLs: transferring	Ordinal	Without assistance = 0, with assistance = 0.5, bedridden = 1
12	ADLs: continence	Ordinal	Without assistance = 0, occasional accidents = 0.5, incontinent = 1
13	ADLs: feeding	Ordinal	Without assistance = 0, with some help=0.5, need feeding= 1
14	Functional limitations: hand behind neck	Ordinal	Both hands = 0, left hand = 0.5, right hand = 0.5, neither hand = 1
15	Functional limitations: hand behind lower back	Ordinal	Both hands = 0, left hand = 0.5, right hand = 0.5, neither hand = 1
16	Functional limitations: able to stand up from sitting	Ordinal	Yes, without using hands = 0; Yes, using hands = 0.5; no = 1
17	Functional limitations: able to pick up a book from the floor	Ordinal	Yes, standing = 0; Yes, sitting = 0.5; no = 1
18	Functional limitations: able to use chopsticks to eat	Binary	Yes = 0, no = 1
19	Visual function	Ordinal	Can see and distinguish = 0, can see only = 0.5, can't see = 1, blind = 1
20	Able to hear	Binary	Yes = 0, no = 1
21	Number of steps used to turn around a 360 degree turn without help	Binary	≥6 steps = 1, <6 steps = 0
22	Number of times suffering from serious illness in the past two years	Binary	Suffering from serious illness in the past two years≥1 time(s) = 1, no serious illness = 0
23	Interviewer rated health	Ordinal	Surprisingly healthy = 0, relatively healthy = 0, moderately ill = 0.5, very ill = 1
24	Hypertension	Binary	Yes = 1, no = 0
25	Diabetes	Binary	Yes = 1, no = 0
26	Heart disease	Binary	Yes = 1, no = 0
27	Stroke or cerebrovascular disease	Binary	Yes = 1, no = 0
28	Bronchitis, emphysema, pneumonia, asthma	Binary	Yes = 1, no = 0
29	Tuberculosis	Binary	Yes = 1, no = 0
30	Cancer	Binary	Yes = 1, no = 0

31	Gastric or duodenal ulcer	Binary	Yes = 1, no = 0
32	Parkinson	Binary	Yes = 1, no = 0
33	Bedsore	Binary	Yes = 1, no = 0
34	Cataract	Binary	Yes = 1, no = 0
35	Glaucoma	Binary	Yes = 1, no = 0
36	Prostate Tumor	Binary	Yes = 1, no = 0

Abbreviations: ADLs = activities of daily living.

Table S2. Baseline variable definitions in the present study

Variable	Questions in the CLHLS questionnaires	Options of questions in the CLHLS questionnaires	Reclassifications of options in the present study
Sex		• male<br>• female	• male<br>• female
Age	Validated age		• Age: years
Marital status	Current marital status?	• currently married and living with spouse<br>• separated<br>• divorced<br>• widowed<br>• never married<br>• don't know<br>• missing	• in marriage: currently married and living with spouse, separated<br>• not in marriage: divorced, widowed, never married<br>• missing: don't know, missing
Residence	Current residence area of interviewee?	Wave 1998:<br>• urban (city and town)<br>• rural<br>Waves 2000, 2002, 2005, 2008, 2011, 2014:<br>• city<br>• town<br>• rural	• urban: city, town<br>• rural: rural
Co-residence	Co-residence?	• with household member(s)<br>• alone<br>• in an institution<br>• missing	• with household member(s)<br>• alone<br>• in an institution<br>• missing
Education	How many years did you attend school?	• years of school<br>• don't know<br>• missing	• No school: years of school = 0<br>• 1 year or more: years of school ≥1<br>• missing: don't know, missing
Current smoking	Do you smoke at the present time?	• yes<br>• no<br>• missing	• current smoking: yes<br>• no smoking at present: no<br>• missing
Current drinking	Do you drink alcohol at the present time?	• yes<br>• no<br>• missing	• current drinking: yes<br>• no drinking at present: no<br>• missing
Current regular exercise	Do you do exercises regularly at present?	• yes<br>• no<br>• missing	• current regular exercise: yes<br>• no regular exercise at present: no<br>• missing
Fresh fruit	How often eat fresh fruit?	Waves 1998, 2000, 2002, 2005:<br>• almost everyday<br>• except winter<br>• occasionally<br>• rarely or never<br>• missing	• Regular intake: almost everyday, except winter/quite often<br>• No regular intake: occasionally, rarely or never<br>• missing: don't know, missing

		Waves 2008, 2011, 2014:<br>• almost everyday<br>• quite often<br>• occasionally<br>• rarely or never<br>• don't know<br>• missing	
Fresh vegetables	How often eat vegetables?	Waves 1998, 2000, 2002, 2005:<br>• almost everyday<br>• except winter<br>• occasionally<br>• rarely or never<br>• missing<br>Waves 2008, 2011, 2014:<br>• almost everyday<br>• except winter<br>• occasionally<br>• rarely or never<br>• don't know<br>• missing	• Regular intake: almost everyday, except winter<br>• No regular intake: occasionally, rarely or never<br>• missing: don't know, missing
Regular intake of meat, fish, eggs, and beans, respectively	How often eat these foods at present, respectively?	Waves 1998, 2000, 2002, 2005:<br>• almost everyday<br>• occasionally<br>• rarely or never<br>• missing<br>Waves 2008, 2011, 2014:<br>• almost everyday<br>• not everyday, but at least once per week<br>• not every week, but at least once per month<br>• not every month, but occasionally<br>• rarely or never<br>• don't know<br>• missing	• Regular intake: almost everyday; not everyday, but at least once per week<br>• No regular intake: occasionally; not every week, but at least once per month; not every month, but occasionally; rarely or never.<br>• missing: don't know, missing
Diabetes, heart diseases, cerebrovascular diseases, respiratory diseases, and cancer, respectively	Are you suffering from these diseases, respectively?	• yes<br>• no<br>• don't know<br>• missing	• yes: yes<br>• no: no<br>• missing: don`t know, missing
Systolic blood pressure (SBP)			SBP (mm Hg)
Diastolic blood pressure (DBP)			DBP (mm Hg)

Abbreviations: CLHLS = Chinese Longitudinal Healthy Longevity Surveys; SBP = systolic blood pressure; DBP = diastolic blood pressure.

Table S3. Distributions of baseline variables with missing data

Variable	Number of missing data	Percentage with missing data (%)
Marital status	4	0.03
Residenc	0	0.00
Co-residence	3	0.02
Education	80	0.63
Current smoking	1	0.01
Current drinking	7	0.05
Current regular exercise	5	0.04
Regular intake of fruit	4	0.03
Regular intake of vegetables	6	0.05
Regular intake of meat	26	0.20
Regular intake of fish	43	0.34
Regular intake of eggs	23	0.18
Regular intake of beans	17	0.13
Diabetes	0	0.00
Heart disease	0	0.00
Cerebrovascular diseases	0	0.00
Respiratory disease	0	0.00
Cancer	0	0.00
SBP (mmHg)	0	0.00
DBP (mmHg)	0	0.00

Participants with missing values were deleted in the main statistical analyses, and we performed multiple imputation for missing values as a sensitivity analysis. Abbreviations as in Table 1

Table S4. Akaike information criterion values for parametric models

Distribution	Akaike information criterion values
weibull	37011.6
loglogistic	37408.2
exponential	37810.0
lognormal	37965.8
gaussian	41429.3
logistic	41582.1

Table S5. Associations of frailty status with overall survival in hypertensive patients after excluding deaths within the first year or first two years

	Frailty status				p for trend [b]
	Robustness	Pre-frailty	Mild frailty	Moderate-severe frailty	
• *Excluding deaths within the first year (n=9244)*					
Number of participants	3159	4352	1299	434	
Number of deaths	1718	2970	1013	377	
Follow-up (PYs)	17914.9	19924.5	4785.5	1281.3	
Mortality rates[a] (95% CI)	9.6 (9.2-10.0)	14.9 (14.4-15.4)	21.2 (20.0-22.3)	29.4 (26.9-31.9)	
Unadjusted TR (95% CI), p	1.00 (ref)	0.71 (0.68-0.74), <0.001	0.53 (0.50-0.56), <0.001	0.40 (0.37-0.43), <0.001	<0.001
Adjusted TR (95% CI), p					
Model 1	1.00 (ref)	0.87 (0.84-0.90), <0.001	0.74 (0.70-0.77), <0.001	0.62 (0.58-0.66), <0.001	<0.001
Model 2	1.00 (ref)	0.88 (0.85-0.91), <0.001	0.74 (0.71-0.78), <0.001	0.62 (0.58-0.67), <0.001	<0.001
Model 3	1.00 (ref)	0.88 (0.85-0.91), <0.001	0.75 (0.72-0.78), <0.001	0.64 (0.59-0.68), <0.001	<0.001
• *Excluding deaths within the first two years (n=7660)*					
Number of participants	2881	3590	932	257	
Number of deaths	1440	2208	646	200	
Follow-up (PYs)	17500.8	18778.5	4236.9	1025.1	
Mortality rates[a] (95% CI)	8.2 (7.8-8.6)	11.8 (11.3-12.2)	15.2 (14.2-16.3)	19.5 (17.1-21.9)	
Unadjusted TR (95% CI), p	1.00 (ref)	0.79 (0.76-0.81), <0.001	0.65 (0.62-0.68), <0.001	0.54 (0.5-0.58), <0.001	<0.001
Adjusted TR (95% CI), p					
Model 1	1.00 (ref)	0.92 (0.89-0.95), <0.001	0.82 (0.79-0.86), <0.001	0.74 (0.69-0.79), <0.001	<0.001
Model 2	1.00 (ref)	0.93 (0.90-0.96), <0.001	0.83 (0.80-0.86), <0.001	0.75 (0.70-0.80), <0.001	<0.001
Model 3	1.00 (ref)	0.93 (0.90-0.96), <0.001	0.83 (0.80-0.87), <0.001	0.75 (0.70-0.80), <0.001	<0.001

[a] Per 100 person-years.
[b] Test for trend on variable containing median value of FI for each group.
Model 1 with adjustment for sex and age.
Model 2 with adjustment for variables in model 1 plus marital status, residence, co-residence, education, systolic BP and diastolic BP.
Model 3 with adjustment for variables in model 2 plus lifestyles (current smoking, current drinking, current regular exercise), and regular intake of foods (fruit, vegetables, meat, fish, eggs, beans).
Abbreviations: PYs = person-year; TR = time ratio; CI = confidence interval; FI = frailty index; BP = blood pressure.

Table S6. Associations of frailty status with overall survival in hypertensive patients after multiple imputation (n = 10626)

	Frailty status				p for trend[b]
	Robustness	Pre-frailty	Mild frailty	Moderate-severe frailty	
Number of participants	3372	5009	1598	647	
Number of deaths	1917	3596	1310	589	
Follow-up (PYs)	18119.7	20577.4	4982.7	1401.2	
Mortality rates[a] (95% CI)	10.6 (10.1-11.0)	17.5 (17.0-18.0)	26.3 (25.1-27.5)	42.0 (39.5-44.6)	
Unadjusted TR (95% CI), p	1.00 (ref)	0.63 (0.61-0.67), <0.001	0.43 (0.41-0.46), <0.001	0.28 (0.26-0.30), <0.001	<0.001
Adjusted TR (95% CI), p					
Model 1	1.00 (ref)	0.82 (0.79-0.86), <0.001	0.66 (0.62-0.70), <0.001	0.49 (0.46-0.53), <0.001	<0.001
Model 2	1.00 (ref)	0.83 (0.79-0.87), <0.001	0.67 (0.63-0.70), <0.001	0.50 (0.46-0.53), <0.001	<0.001
Model 3	1.00 (ref)	0.84 (0.80-0.87), <0.001	0.68 (0.64-0.72), <0.001	0.51 (0.48-0.55), <0.001	<0.001

[a] Per 100 person-years.

[b] Test for trend on variable containing median value of FI for each group.

Model 1 with adjustment for sex and age.

Model 2 with adjustment for variables in model 1 plus marital status, residence, co-residence, education, systolic BP and diastolic BP.

Model 3 with adjustment for variables in model 2 plus lifestyles (current smoking, current drinking, current regular exercise), and regular intake of foods (fruit, vegetables, meat, fish, eggs, beans).

Abbreviations: PYs ==person-year; TR = time ratio; CI = confidence interval; BP = blood pressure.

Table S7. Associations of frailty status with overall survival in hypertensive patients in considering the losses censored at the two time points: median (3.38 years) and the end of follow-up (10.00 years) (n = 12575)

	Frailty status				p for trend[b]
	Robustness	Pre-frailty	Mild frailty	Moderate-severe frailty	
• *Considering the losses censored at the median of the study*					
Number of participants	3919	5893	1956	807	
Number of deaths	1905	3542	1301	579	
Follow-up (PYs)	19962.2	23533.8	6197.0	1960.7	
Mortality rates[a] (95% CI)	9.5 (9.1-10.0)	15.1 (14.6-15.5)	21.0 (20.0-22.0)	29.5 (27.5-31.5)	
Unadjusted TR (95% CI), p	1.00 (ref)	0.67 (0.64-0.70), <0.001	0.49 (0.46-0.52), <0.001	0.36 (0.33-0.38), <0.001	<0.001
Adjusted TR (95% CI), p					
Model 1	1.00 (ref)	0.84 (0.81-0.88), <0.001	0.72 (0.69-0.76), <0.001	0.62 (0.57-0.66), <0.001	<0.001
Model 2	1.00 (ref)	0.85 (0.82-0.89), <0.001	0.73 (0.69-0.77), <0.001	0.62 (0.57-0.66), <0.001	<0.001
Model 3	1.00 (ref)	0.86 (0.83-0.90), <0.001	0.74 (0.70-0.78), <0.001	0.63 (0.59-0.68), <0.001	<0.001
• *Considering the losses censored at the end of the study*					
Number of participants	3919	5893	1956	807	
Number of deaths	1905	3542	1301	579	
Follow-up (PYs)	23755.5	29948.6	8639.8	3092.7	
Mortality rates[a] (95% CI)	8.0 (7.7-8.4)	11.8 (11.5-12.2)	15.1 (14.3-15.8)	18.7 (17.3-20.1)	
Unadjusted TR (95% CI), p	1.00 (ref)	0.68 (0.64-0.71), <0.001	0.53 (0.49-0.57), <0.001	0.42 (0.38-0.47), <0.001	<0.001
Adjusted TR (95% CI), p					
Model 1	1.00 (ref)	0.87 (0.82-0.92), <0.001	0.82 (0.76-0.88), <0.001	0.78 (0.71-0.86), <0.001	<0.001
Model 2	1.00 (ref)	0.88 (0.84-0.93), <0.001	0.81 (0.76-0.87), <0.001	0.77 (0.70-0.84), <0.001	<0.001
Model 3	1.00 (ref)	0.90 (0.85-0.95), <0.001	0.83 (0.77-0.89), <0.001	0.78 (0.71-0.86), <0.001	<0.001

[a] Per 100 person-years.
[b] Test for trend on variable containing median value of FI for each group.
Model 1 with adjustment for sex and age.
Model 2 with adjustment for variables in model 1 plus marital status, residence, co-residence, education, systolic BP and diastolic BP.
Model 3 with adjustment for variables in model 2 plus lifestyles (current smoking, current drinking, current regular exercise), and regular intake of foods (fruit, vegetables, meat, fish, eggs, beans).
Abbreviations: PYs = person-year; TR = time ratio; CI = confidence interval; BP = blood pressure.

Table S8. Associations of frailty status with overall survival in hypertensive patients with two BP measurements (n=1164)

	Frailty status				p for trend[b]
	Robustness	Pre-frailty	Mild frailty	Moderate-severe frailty	
Number of participants	428	538	148	50	
Number of death participants	181	337	114	46	
Follow-up (PYs)	2256.6	2310.4	501.7	119.5	
Mortality rates[a] (95% CI)	8.0 (6.9-9.1)	14.6 (13.1-16.0)	22.7 (19.1-26.4)	38.5 (29.8-47.2)	
Unadjusted TR (95% CI), p	1.00 (ref)	0.60 (0.51-0.69), <0.001	0.40 (0.32-0.48), <0.001	0.25 (0.19-0.32), <0.001	<0.001
Adjusted TR (95% CI), p					
Model 1	1.00 (ref)	0.79 (0.69-0.90), 0.001	0.70 (0.58-0.83), <0.001	0.41 (0.32-0.52), <0.001	<0.001
Model 2	1.00 (ref)	0.80 (0.70-0.91), 0.001	0.69 (0.58-0.82), <0.001	0.41 (0.32-0.52), <0.001	<0.001
Model 3	1.00 (ref)	0.82 (0.71-0.93), 0.003	0.72 (0.60-0.86), <0.001	0.43 (0.34-0.55), <0.001	<0.001

[a] Per 100 person-years.
[b] Test for trend on variable containing median value of FI for each group.
Model 1 with adjustment for sex and age.
Model 2 with adjustment for variables in model 1 plus marital status, residence, co-residence, education, systolic BP and diastolic BP.
Model 3 with adjustment for variables in model 2 plus lifestyles (current smoking, current drinking, current regular exercise), and regular intake of foods (fruit, vegetables, meat, fish, eggs, beans).
Abbreviations: PYs = person-year; TR = time ratio; CI = confidence interval; BP = blood pressure.

Figure S1. The spatial distributions of the study population

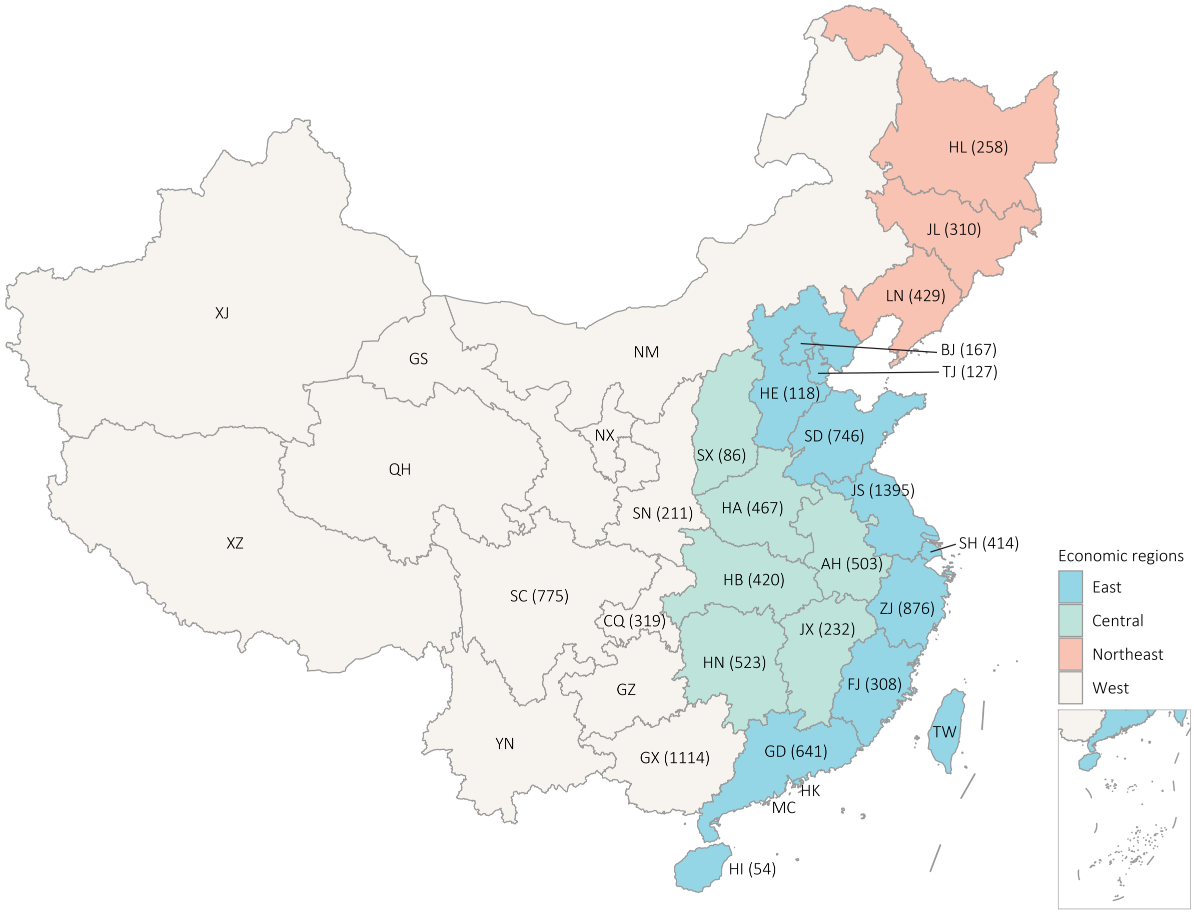

In the present study, province with the most study participants was Jiangsu (n = 1395), followed by Guangxi, Zhejiang, Sichuan, Shandong, Guangdong, Hunan, Anhui, Henan, Liaoning, Hubei, Shanghai, Chongqing, Jilin, Fujian, Heilongjiang, Jiangxi, Shaanxi, Beijing, Tianjin, Hebei, Shanxi and Hainan.

Figure S2. Distributions of frailty index among the study participants

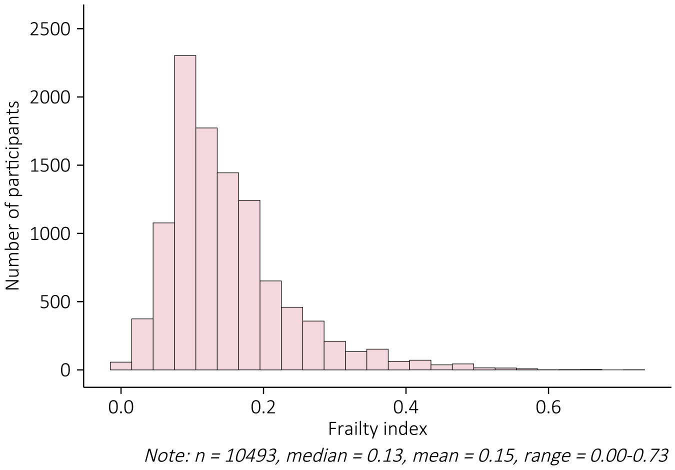

Supplement: Supplementary file 1 — Supplementary Material 1. [file 12889_2024_18989_MOESM1_ESM.pdf]
